# Chromosomal islands of *Streptococcus pyogenes* and related streptococci: molecular switches for survival and virulence

**DOI:** 10.3389/fcimb.2014.00109

**Published:** 2014-08-12

**Authors:** Scott V. Nguyen, William M. McShan

**Affiliations:** ^1^Department of Microbiology and Immunology, The University of Oklahoma Health Sciences CenterOklahoma City, OK, USA; ^2^Department of Pharmaceutical Sciences, The University of Oklahoma Health Sciences CenterOklahoma City, OK, USA

**Keywords:** *Streptococcus pyogenes*, group A streptococcus, phage-like chromosomal islands, SpyCI, mutator phenotype

## Abstract

*Streptococcus pyogenes* is a significant pathogen of humans, annually causing over 700,000,000 infections and 500,000 deaths. Virulence in *S. pyogenes* is closely linked to mobile genetic elements like phages and chromosomal islands (CI). *S. pyogenes* phage-like chromosomal islands (SpyCI) confer a complex mutator phenotype on their host. SpyCI integrate into the 5′ end of DNA mismatch repair (MMR) gene *mutL*, which also disrupts downstream operon genes *lmrP, ruvA*, and *tag*. During early logarithmic growth, SpyCI excise from the bacterial chromosome and replicate as episomes, relieving the mutator phenotype. As growth slows and the cells enter stationary phase, SpyCI reintegrate into the chromosome, again silencing the MMR operon. This system creates a unique growth-dependent and reversible mutator phenotype. Additional CI using the identical attachment site in *mutL* have been identified in related species, including *Streptococcus dysgalactiae* subsp. *equisimilis, Streptococcus anginosus, Streptococcus intermedius, Streptococcus parauberis*, and *Streptococcus canis*. These CI have small genomes, which range from 13 to 20 kB, conserved integrase and DNA replication genes, and no identifiable genes encoding capsid proteins. SpyCI may employ a helper phage for packaging and dissemination in a fashion similar to the *Staphylococcus aureus* pathogenicity islands (SaPI). Outside of the core replication and integration genes, SpyCI and related CI show considerable diversity with the presence of many indels that may contribute to the host cell phenotype or fitness. SpyCI are a subset of a larger family of streptococcal CI who potentially regulate the expression of other host genes. The biological and phylogenetic analysis of streptococcal chromosomal islands provides important clues as to how these chromosomal islands help *S. pyogenes* and other streptococcal species persist in human populations in spite of antibiotic therapy and immune challenges.

## Introduction

*Streptococcus pyogenes* is a significant human pathogen, annually causing over 700,000,000 infections and 500,000 deaths (Carapetis et al., 2005). Genome sequencing has revealed that prophages and other mobile genetic elements are important features of *Streptococcus pyogenes* (group A streptococcus) chromosomes, sometimes contributing up to 10% of the total DNA (Desiere et al., 2001; Ferretti et al., 2001; Banks et al., 2002; Canchaya et al., 2002). These genome prophages follow a typical lambdoid gene arrangement, with sequentially organized modules for integration and lysogeny, DNA replication, transcriptional regulation, DNA packaging and head assembly, tail and tail fiber assembly, and lysis (Desiere et al., 2001; Banks et al., 2002; Canchaya et al., 2002; Brussow et al., 2004). In *S. pyogenes* and many other pathogens, these essential phage genes are often followed by one or more virulence genes such as toxins (Brussow et al., 2004). Numerous genes on *S. pyogenes* chromosomes are the targets for site-specific integration by these mobile genetic elements, and for some of these genes, integration has the potential to interrupt or alter their transcription (McShan and Ferretti, 2007). Of these targeted genes, one location stands out both for its frequency of occupation by a chromosomal island (CI) as well as the potential phenotypic impact integration would have on the cell: the operon encoding the genes for DNA mismatch repair (MMR). We have characterized phage-like CI in *S. pyogenes* that integrate into MMR gene *mutL*, silencing this gene and the other downstream genes of the operon (Scott et al., 2008, 2012). The integration of the *S. pyogenes* Chromosomal Island M1 (SpyCIM1) into the chromosome induces a complex mutator phenotype that results from the interruption of the operon and downstream DNA repair genes (Scott et al., 2008, 2012). Largely due to the extensive and ongoing efforts to sequence the genomes of many species of bacteria, additional islands integrated into *mutL* also have been identified in other *Streptococcus* species. This review will examine the CI identified so far, their known or potential impacts on host phenotype and survival, and implications for the evolution of this group and their host bacteria. A note concerning nomenclature: when referred to collectively as a group, *S. pyogenes* phage-like CI are referred to as SpyCI, but a specific CI is identified so to associated it with a particular strain or isolate (e.g., SpyCIM1, SpyCIM49, etc.). The same convention is applied to CI from other streptococcal species.

## SpyCIM1 and the host mutator phenotype

Typically, site-specific recombination occurs between bacterial and phage genomes such that the transcription of the targeted host gene is unimpeded by the presence of the prophage (Fouts, 2006). This maintenance of gene function is accomplished by two factors: (1) duplication of the host DNA sequence at the site of crossover by a portion of the phage DNA and (2) integration at the 3′ end of the targeted gene so that the duplication can complete the original bacterial ORF (Fouts, 2006; Louie et al., 2007; McShan and Ferretti, 2007). By contrast, integration into the 5′ end or the middle of a gene could result in the disruption of normal transcription with a concomitant loss of gene function. Occasional examples of prophages altering the expression of host genes have been reported in *Escherichia coli* and *Staphylococcus aureus* (Mason and Allen, 1975; Lee and Iandolo, 1986; Thomas and Drabble, 1986; Coleman et al., 1991; Campbell et al., 1992), but these occurrences have been notable in part because of their rarity. By contrast, genome sequencing has revealed that *S. pyogenes* prophages frequently target attachment sites positioned at the promoter or 5′ end of genes that, following integration, potentially could alter gene expression, or create polar mutations (McShan and Ferretti, 2007). Of these mobile genetic elements with the potential to alter gene expression, the phage-like chromosomal island that frequently targets and regulates the DNA mismatch repair (MMR) operon of *S. pyogenes* is perhaps the most remarkable.

MMR has been extensively studied in *E. coli* where the system recognizes base pair mismatches in nascent, hemimethylated DNA and directs strand-specific repair. The newly synthesized unmethylated DNA strand, which contains the mismatch, is cleaved by the MMR system and repair is initiated by re-synthesis of the cleaved region. Gram-positive bacteria and eukaryotes do not rely upon methylation for strand recognition, probably instead relying upon modification of the beta clamp of DNA polymerase III for strand discrimination (Li, 2008). LeClerc and co-workers were the first to observe that the mutator phenotype was present in wild populations of *E. coli* and *Salmonella enterica* at unexpectedly high frequencies and that these phenotypes mapped to mutations in MMR genes (LeClerc et al., 1996). It was subsequently found that mutators were present in both pathogenic and non-pathogenic *E. coli*, suggesting that this trait conferred a selective advantage upon the cell in spite of the risk of increased frequencies of deleterious mutations (Matic et al., 1997). Subsequent studies showed that MMR mutants are frequently isolated from clinical strains of many species. For example, antibiotic treatment of *Pseudomonas aeruginosa* infections in cystic fibrosis patients correlates with the rapid appearance of drug-resistant MMR mutants (LeClerc and Cebula, 2000; Oliver et al., 2002). Other examples have been found in *Neisseria meningitis, Helicobacter pylori, Haemophilus influenzae*, and *Staphylococcus aureus* (Bjorkholm et al., 2001; Richardson et al., 2002; Bayliss et al., 2004; Prunier and Leclercq, 2005; Trong et al., 2005). The frequency of such mutator strains is often high: for example, 20% of *P. aeruginosa* strain from cystic fibrosis patients and over 50% of epidemic-associated serogroup A *N. meningitis* strain are mutators (Oliver et al., 2002; Richardson et al., 2002). The MMR system can act as a barrier for genetic diversity and bacteriophage transduction, thus inhibition of MMR removes this barrier and promotes diversification through homeologous recombination (Limia et al., 1998; Kataja et al., 1999; Matic et al., 2000). However, in all of these species, the MMR defects result from mutations that render *mutS* or *mutL* permanently defective. The resulting mutator phenotype is a double-edged sword, however. The advantages gained by a cell in rapidly acquiring favorable mutations like antibiotic resistance are balanced by the possibility of deleterious mutations arising that diminishes cell viability. In *S. pyogenes*, a remarkable solution has evolved to achieve a mutator phenotype while minimizing the potential risks: a growth-dependent molecular switch controlled by a CI, which allows the cells to be phenotypically wild type when resources are abundant but switching to a mutator phenotype when facing challenges like limited nutrient availability.

**Figure 1:**
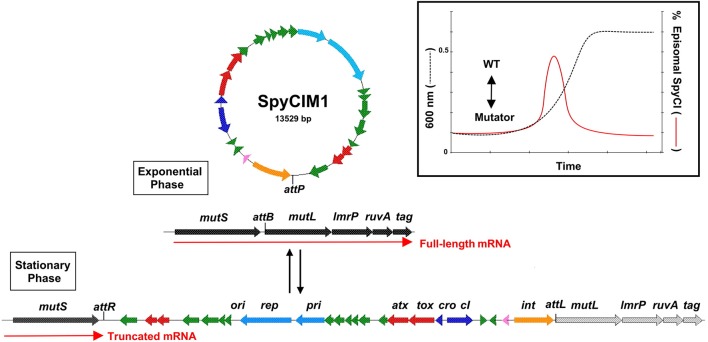
**SpyCIM1 regulates the MMR operon through dynamic site-specific excision and integration**. The molecular switch controlled by SpyCIM1 is shown. The MMR operon of *S. pyogenes* is comprised of genes encoding DNA mismatch repair (*mutS* and *mutL*), multidrug efflux (*lmrP*), a Holliday-junction resolvase (*ruvA*), and base excision repair (*tag*). During exponential phase, SpyCIM1 excises from the chromosome, circularizes, and replicates as an episome, restoring transcription of the entire MMR operon (WT). Excision and mobilization occurs early in logarithmic growth in response to yet unknown cellular signals (Insert; adapted from Scott et al., 2008). As logarithmic growth continues, SpyCIM1 re-integrates into *mutL* at *attB*, and by the time the culture reaches stationary phase, the integration process has completed, again blocking transcription of the MMR operon. WT, Wild type phenotype associated with unimpeded expression of the MMR operon. Color key of predicted gene functions: Green, genes of unknown function; red, possible toxin-antitoxin maintenance genes; light blue, DNA replication; dark blue, control of lysogeny; pink, transmembrane peptide; orange, site-specific integrase.

The MMR operon of *S. pyogenes* M1 strain SF370 (Figure 1) is composed of the genes *mutS, mutL, lmrP, ruvA*, and *tag*, which encode MMR, a multidrug efflux pump of the major facilitator family, a Holliday junction resolvase, and base excision repair glycosylase, respectively, (Ferretti et al., 2001). These genes are grouped on a polycistronic mRNA that is controlled by a single promoter upstream of *mutS*. Analysis of the M1 genome showed that the phage-like chromosomal island SpyCIM1 was integrated between *mutS* and *mutL*, and this integration was subsequently found to interrupt the expression of *mutL* and the downstream genes (Scott et al., 2008, 2012). SpyCIM1 is not a static element, permanently residing in the bacterial genome like a typical prophage, but rather a dynamic element that excises from chromosome during early logarithmic growth and replicates as a circular episome (Scott et al., 2008). As the bacterial population reaches the end of logarithmic phase and enters stationary phase, SpyCIM1 re-integrated into its unique attachment site (*attB*) at the beginning of *mutL* (Figure 1, insert). Thus, SpyCIM1 acts as a growth-dependent molecular switch to control the expression of MMR. The outcome of this switch is that the *S. pyogenes* cell alternates between a mutator and wild type phenotype in response to growth: during rapid cell division and DNA replication, the integrity of the genome is maintained by an active MMR system while during stationary phase or other periods of infrequent cell division, mutations may accumulate at a higher rate.

This molecular switch controls additional operon genes downstream of *mutL*. The next gene, *lmrP*, encodes a putative multidrug resistance efflux pump (MDR) of the major facilitator family (Bolhuis et al., 1995; Putman et al., 2001). In *Lactococcus lactis*, the gene was characterized as an ATP dependent pump that extrudes multiple drugs across the membrane, preventing toxic accumulations of these chemicals in the bacteria (Bolhuis et al., 1995). Presently, the natural substrate for LmrP in *S. pyogenes* is unknown, as is why a multidrug efflux pump is transcriptionally linked with a DNA repair operon in group A and related species of streptococci (Supplemental Table 1). However, the ability to regulate expression of this gene may have selective advantage for the streptococcus. In *Listeria monocytogenes* an LmrP homolog (*mdrM*) exists whose expression or inhibition can control the magnitude of the host cytosolic response to infection, and loss of MdrM protein function leads to a 3-fold reduction in IFN-β response to infection (Crimmins et al., 2008). It may be that inhibition of LmrP expression in *S. pyogenes* similarly provides a mechanism of innate immunity avoidance. Indeed, if true, SpyCI regulation of the LmrP in *S. pyogenes* may have a large biological impact given how a MDR can influence multiple processes in a cell by removal of toxic or inhibitory substances.

The next gene on the polycistronic message, *ruvA*, encodes an ATP-dependent helicase that promotes branch migration of Holliday junctions during homologous genetic recombination and recombinant repair of damaged DNA. The loss of RuvA function leads to increased sensitivity to UV damage as irradiation induced DNA lesions lead to arrested replication forks (Iwasaki et al., 1989; Tsaneva et al., 1992; Kaplan and O'Donnell, 2006). This increase in sensitivity to UV irradiation is clearly observed in *S. pyogenes* strains carrying SpyCI (Scott et al., 2008, 2012).

The last gene on the operon, *tag*, encodes a 3-methyladenine DNA glycosylase I which is involved in base excision repair (Bjelland et al., 1993). This enzyme is important in recognizing and purging aberrant and modified bases from damage induced by DNA damaging elements such as the alkylating agent ethyl methanesulfonate (Wyatt et al., 1999). Loss of the 3-methyladenine DNA glycosylase greatly increases the spontaneous mutation rate associated with single nucleotide substitution (Kaasen et al., 1986; Bjelland et al., 1993; Wyatt et al., 1999). The loss of gene expression from *mutL* to *tag* causes the cell to exhibit a complex mutator phenotype that impacts several DNA repair or maintenance systems (Scott et al., 2008, 2012). Indeed, the silencing of this operon may necessarily need to be reversed occasionally to maintain cell viability, given our observation that permanent loss of the ability to excise from the bacterial chromosome lead to the use of a new promoter to express these genes in M5 strain Manfredo (Scott et al., 2012).

SpyCI are frequent genetic elements in *S. pyogenes* genomes (Table 1). M serotypes associated with SpyCI carriage currently include M1, M2, M4, M5, M6, M18, M25, M28, M31, M37, M49, M53, M59, M78, and M123 (Scott et al., 2008, 2012; Suvorov et al., 2009). Currently, it is not known whether SpyCI infect only a subset of *S. pyogenes* serotypes, perhaps defined by surface targets for phage attachment, or whether most serotypes may serve as SpyCI hosts and the current sample size is simply too small. Other factors such as phage immunity proteins or the dependence of SpyCI on helper phages with limited host ranges may also influence the dissemination of these chromosomal islands. At least from the standpoint of integration, virtually all group A streptococci could serve as a host for SpyCI since the *attB* DNA sequence at the beginning of *mutL* is highly conserved.

**Table 1:** ***S. pyogenes* strains with SpyCI integrated into DNA MMR gene *mutL* that have been identified by genome sequencing**.

**Strain**	**Serotype**	**Chromosomal island**	**GenBank Accession**
SF370	M1	SpyCIM1	AE004092
MGAS10270	M2	SpyCIM2	CP000260
MGAS10750	M4	SpyCIM4	CP000262
Manfredo	M5	SpyCIM5	AM295007
GA40377	M5	SpyCIM5 GA40377	AWTK01000000
GA41046	M5	SpyCIM5 GA41046	AWWB01000000
UTMEM-1	M5	SpyCI UTMEM-1	NZ_AVCF00000000
UTSW-2	M5	SpyCI UTSW-2	NZ_AVCG00000000
MGAS10394	M6	SpyCIM6	CP000003
GA19700	M6	SpyCIM6 GA19700	AWTF01000000
GA41039	M6	SpyCIM6 GA41039	AWTG01000000
GA41208	M6	SpyCIM6 GA41208	AWTH01000000
T25_3_	M25	SpyCIM25	(Unpublished)
MGAS 6180	M28	SpyCIM28	CP000056
GA03747	M49	SpyCIM49 GA03747	AWUB01000000
GA16797	M49	SpyCIM49 GA16797	AWUD01000000
GA40634	M49	SpyCI GA40634	NZ_AURU00000000
Alab49	M53	SpyCIM53	CP003068
MGAS1882	M59	SpyCIM59	CP003116
MGAS15252	M59	SpyCIM59.1	CP003121

**Figure 2:**
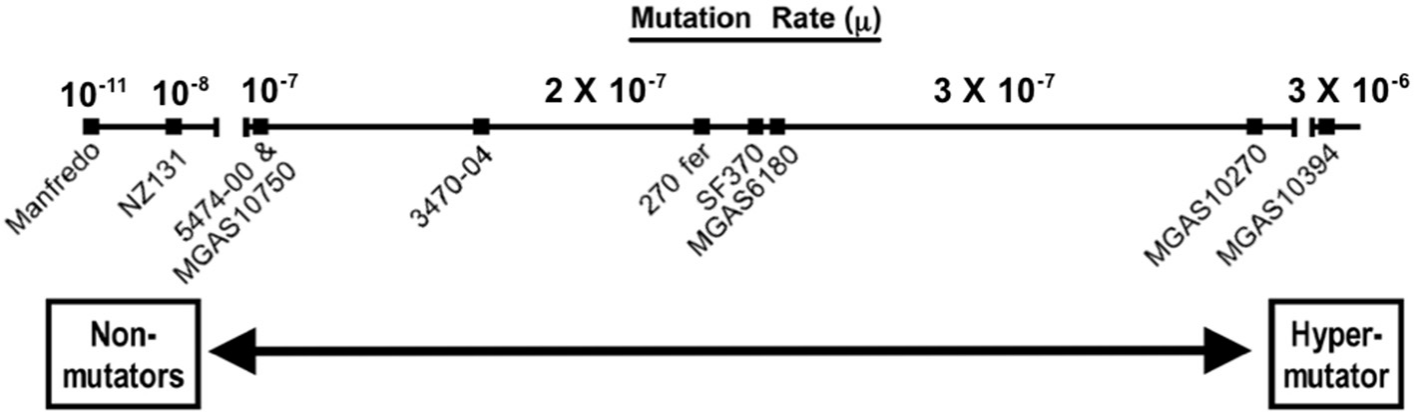
**The mutation rates of SpyCI^+^*S. pyogenes* genome and clinical strains**. Strains harboring SpyCI integrated into *mutL* have phenotypes that range from non-mutator in Manfredo, where a novel CI promoter in the defective integrase gene rescues expression of *mutL*, to a hypermutator in M6 genome strain MGAS10394. Strain NZ131 does not carry a SpyCI and is presented as a wild type strain with regards to the MMR operon. The mutation rate is calculated as mutations per generation. The figure was drawn using the data from Scott et al. (2012).

In general, the presence of a SpyCI in a given *S. pyogenes* strain correlates with a higher mutation rate and UV sensitivity when compared to strains lacking this chromosomal island (Scott et al., 2012). Different SpyCI^+^ strains do show a range of mutation rates, however (Figure 2). When compared to SpyCI-free strain NZ131, genome strains with the chromosomal island showed mutation rates that ranged between 5 and 167 times higher. Similarly, resistance to UV irradiation also showed a range of sensitivities. This strain-to-strain variation may reflect differences in SpyCI regulation that determine whether the chromosomal island tends to be integrated into *mutL* or excised as an episome (i.e., how frequently the MMR operon is transcriptionally active). Variations in the operator controlling the repressor and antirepressor may play a role in this decision to remain integrated or extrachromosomal as well as variations in other DNA repair genes that affect the overall cell mutation rate (Scott et al., 2012). The one exception to this general trend was found in serotype M5 strain Manfredo, which has a 128 bp deletion in the SpyCI integrase gene that renders it inactive but a mutation rate that was a 1000-fold lower than NZ131 (Figure 2). So, in spite of the fact that SpyCIM5 was permanently integrated into the Manfredo chromosome, this strain was wild type for the MMR operon. This paradox was resolved by the discovery of a novel promoter within the SpyCIM5 integrase pseudogene that rescued the expression of *mutL* and the downstream genes. Interestingly, expression from this novel promoter was depressed by mitomycin C treatment, which was in contrast to the activation of the MMR in SF370 and other strains with a SpyCI capable of excision from *mutL*. It remains unknown whether this apparent mechanism of gene expression control is the result of natural selection or merely a circumstantial byproduct of evolution of this compensatory promoter (Scott et al., 2012).

Comparisons of mutation rates between strains possessing or lacking SpyCI is informative, but the conclusions are inferential since other genes not in this operon may influence the observed final phenotype. Therefore, to directly assess the effect of SpyCIM1 carriage alone, the chromosomal island was cured from the M1 SF370 genome to create an isogenic derivative, and comparisons of the two strains show that island integration is responsible for a 200-fold increased mutation rate, increased sensitivity to ethidium bromide, increased UV irradiation sensitivity, and higher rates of single point mutations (Euler et al., submitted). As the island excises in response to growth, this dynamic regulation of the operon allows the organism to maintain genetic fidelity in optimal conditions while selectively increasing mutation rates during stressful conditions (Scott et al., 2008).

## Conservation and diversity of SpyCI genes

SpyCI and other phage-like chromosomal islands may have originated from defective prophages, but their biology suggests an even more complex origin. The defining gene of SpyCI is the integrase (*int*), whose expression and regulation controls the molecular switch for the MMR operon. Within the known SpyCI, *int* is highly conserved at both the gene and protein level (Scott et al., 2008, 2012). The SpyCI integrase genes form a distinct group within the *S. pyogenes* prophage integrases (McShan, 2005), although the integrase encoded by *S. pyogenes* strain NZ131 prophage NZ131.1 is 62% identical to the SpyCIM1 integrase at the amino acid level, suggesting that these genes may have had a recent common origin (McShan et al., 2008). The integration site for prophage NZ131.1 is near the promoter of hypothetical protein Spy49_0371 (McShan et al., 2008), which is unrelated to the MMR operon.

Typical for prophages and phage-like CI, two genes encoding predicted DNA-binding proteins are found upstream of *int* and arranged in opposite orientations flanking a probable operator site. These genes, which encode the predicted repressor and antirepressor, are the likely candidates for the control of SpyCI integration and excision. Excision of SpyCI can be induced by mitomycin C treatment (Scott et al., 2008, 2012), suggesting that the mostly uncharacterized *S. pyogenes* SOS DNA repair pathway can trigger SpyCI excision and may lead to packaging by a helper prophage for dissemination to new host cells (Nguyen, unpublished observations). However, the cellular signals that induce the normal cycle of SpyCI excision and re-integration during growth, which presumably is triggered by repressor cleavage, are yet unknown. Interestingly, these genes and their operators vary between the different SpyCI, and these differences may contribute to the range of mutation rates seen between the different *S. pyogenes* genome strain that harbor these CI (Scott et al., 2012). That is, SpyCI repressors that are more sensitive to cleavage would favor the episomal form of the SpyCI and the appearance of the wild type phenotype with respect to MMR while repressors that are more stable would favor integration and the mutator phenotype. The operator DNA sequences of SpyCIM1, SpyCIM2, SpyCIM28, and SpyCIM53 are all nearly identical while the lysogeny module of the hypermutator M6 strain MGAS10394 is essentially the same as the emerging hypervirulent strain MGAS15252 (Fittipaldi et al., 2012; Scott et al., 2012). As we previously pointed out, the SpyCI controlled mutator phenotype in MGAS15252 may contribute to the many striking phenotypic changes that are associated with this strain, which include enhanced transmission by skin contact, significantly impaired ability to grow in saliva, and a tendency not to colonize the oropharynx (Scott et al., 2012).

Other SpyCI genes are conserved, forming part of the core set of genes that identify these mobile genetic elements. The predicted primase and replicase genes (Figure 1) are closely related to homologs from *Streptococcus thermophilus* plasmid pSt106, which are essential for plasmid replication (Geis et al., 2003). While the molecular details of these genes in SpyCI DNA replication and extrachromosomal maintenance remain to be determined, their high degree of conservation within these CI argues their essential role (Scott et al., 2008), presumable to ensure DNA replication of episomal SpyCI. Other SpyCI genes show various degrees of conservation. One universally conserved gene, which was not originally annotated due to its small size, is positioned immediately upstream of *int* and encodes a small transmembrane domain peptide of unknown function (Figure 1). If this ORF really represents an expressed gene, then its product has the potential to alter the surface properties of the streptococcal cell, potentially altering its antigenicity, functioning as an environmental sensor, or providing a protection mechanism against lytic phages by interfering with their attachment. Other genes, based upon homology with known homologs, may function like a toxin-antitoxin pair to prevent SpyCI eliminations (labeled *tox* and *atx* in Figure 1). None of the SpyCI-encoded genes include an identifiable DNA polymerase or a terminase subunit, which plays an important role in the helper phage packaging process of the *Staphylococcus aureus* pathogenicity islands (SaPI) (Novick et al., 2010). Most of the remaining ORFs in the SpyCI encode products of unknown function, and much variation exists between the individual members (Scott et al., 2008). The driving forces behind the genetic diversity of SpyCI from different *S. pyogenes* isolates are poorly understood at present.

## Mismatch repair chromosomal islands of other *streptococcus* species

The transcriptional linking of *mutS* and *mutL* is not universal in prokaryotes, although analysis of publicly available genomes shows that many streptococcal species do group these genes as a unit. This group includes, in addition to *S. pyogenes, Streptococcus mutans, Streptococcus equi* subspecies *equi* and subsp. *zooepidemicus, Streptococcus agalactiae, Streptococcus uberis*, and *Streptococcus thermophilus* (Table 2). Interestingly, *Streptococcus pneumoniae* does not follow this pattern, encoding MMR genes *hexA* and *hexB* at distant sites on its chromosome. Genes *ruvA* and *tag* are positioned near *hexB* on the *S. pneumoniae* chromosome and are probably co-transcribed with each other but not with *hexB*. The gene composition of the operon found in *S. pyogenes* and other related streptococci may represent an instance where evolution has selected for an arrangement that simplifies the control of expression for several housekeeping genes. This arrangement, however, has allowed mobile genetic elements like the SpyCI to assume a unique regulatory role.

**Table 2:** **Chromosomal islands identified in other streptococcal species that target *mutL* or other genes for integration**.

**Species**	**Strain**	**CI**	***attB***	**Gene target**	**GenBank accession**
*S. agalactiae*	ATCC 13813	SagCI	*rpsD*	3′	AEQQ01000005
*S. anginosus*	1505	SanCI	*mutL*	5′	NZ_BASW00000000
*S. anginosus*	C238	SanCI	*mutL*	5′	CP003861
*S. anginosus*	F0211	SanCI	*mutL*	5′	AECT00000000
*S. anginosus*	J4206	SanCI	*mutL*	5′	KC617870
*S. anginosus*, subsp. *whileyi*	CCUG 39159	SanCI	*mutL*	5′	AICP00000000
*S. anginosus*, subsp. *whileyi*	MAS624	SanCI	*mutL*	5′	AP013072
*S. canis*	FSL Z3-227	ScaCI	*mutL*	5′	NZ_AIDX00000000
*S. dysgalactiae*, subsp. *equisimilis*	167	SeqCI	*mutL*	5′	AP012976
*S. dysgalactiae*, subsp. *equisimilis*	SK1249	SeqCI	*mutL*	5′	AFIN01000000
*S. intermedius*	ATCC 27335	SinCI	*mutL*	5′	NZ_ATFK00000000
*S. intermedius*	F0413	SinCI	*mutL*	5′	AFXO00000000
*S. intermedius*	JTH08	SinCI	*mutL*	5′	AP010969
*S. intermedius*	SK54	SinCI	*mutL*	5′	NZ_AJKN00000000
*S. mitis*	11/5	SmiCI	*uvrA*	5′	AQTT01000001
*S. mitis*	B6	SmiCI	*manA*	5′	FN568063
*S. mitis*	SK569	SmiCI	*urvA*	5′	NZ_AFUF01000000
*S. mitis*	SK616	SmiCI	*urvA*	5′	AICR00000000
*S. mitis*	SK1073	SmiCI	*uvrA*	5′	AFQT01000001
*S. mitis*	SK1080	SmiCI	*uvrA*	5′	
*S. parauberis*	KCTC 11537	SpaCI	*mutL*	5′	CP002471
*S. parauberis*	KRS-02083	SpaCI	*mutL*	5′	NZ_ALYM00000000
*S. parauberis*	KRS-02109	SpaCI	*mutL*	5′	NZ_ALWR00000000
*S. pneumoniae*	2071004	SpnCI	*uvrA*	5′	ALBJ00000000
*S. pneumoniae*	2080913	SpnCI	*uvrA*	5′	ALBL00000000
*S. pneumoniae*	GA14688	SpnCI	*uvrA*	5′	AIKQ01000003
*S. pneumoniae*	GA17719	SpnCI	*uvrA*	5′	AILO01000005
*S. pneumoniae*	Hungary 19A-6	SpnCI	*uvrA*	5′	NC_010380
*S. pseudo-pneumoniae*	ATCC BAA-960	SpsCI	*uvrA*	5′	AICS01000001
*S.* species	M334	CI	*uvrA*	5′	PRJNA62529
*S. suis*	D9	SsuCI	*recF*	5′	NC_017620
*S. thermophilus*	CNRZ1066	SthCI	*metE*	5′	NC_006449

Except for SagCI integration into the 3 ' end of rpsD, all CI listed integrated into the 5 ' end of their targeted genes and thus could alter their transcription. Gene key: manA—alpha-1,2-mannosidase; metE—methionine synthase; mutL—DNA MMR protein MutL; recF—recombination protein RecF; rpsD—30S ribosomal protein S4; uvrA—UvrABC endonuclease subunit.

**Figure 3:**
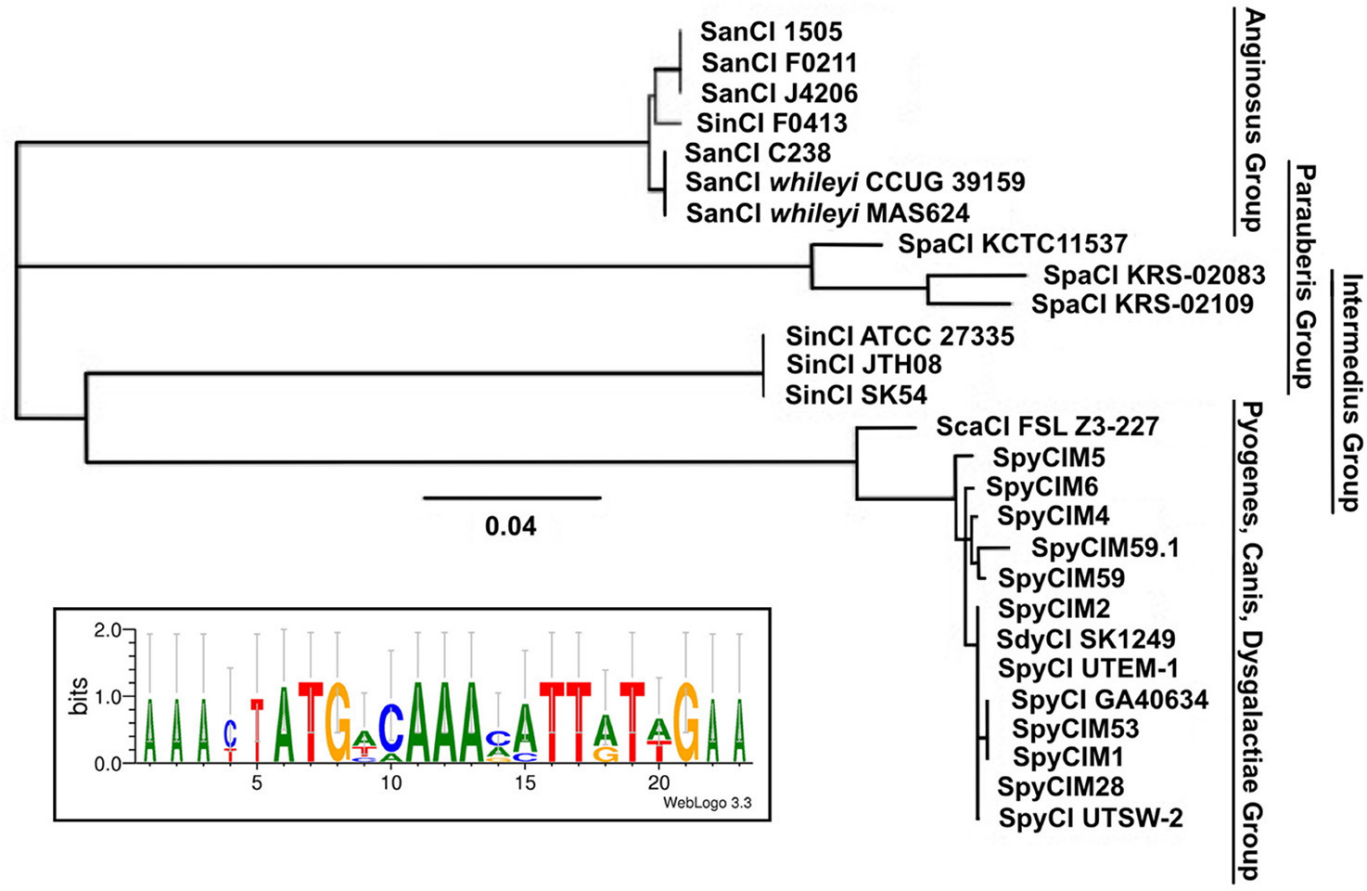
**Phylogenetic tree of the streptococcal CI integrases that target MMR gene *mutL***. Analysis of the chromosomal island integrases amino acid sequences that target *mutL* was used to construct a phylogenetic tree showing the four known major groups. The insert shows the consensus alignment of the *mutL*. Strain details are given in Tables 1, 2. The proteins encoded by the CI integrase genes were aligned and the phylogenetic tree created using Geneious v. 6.1.7 (Drummond et al., 2012). The consensus of the *mutL* attachment site was created using WebLogo (Crooks et al., 2004).

**Figure 4:**
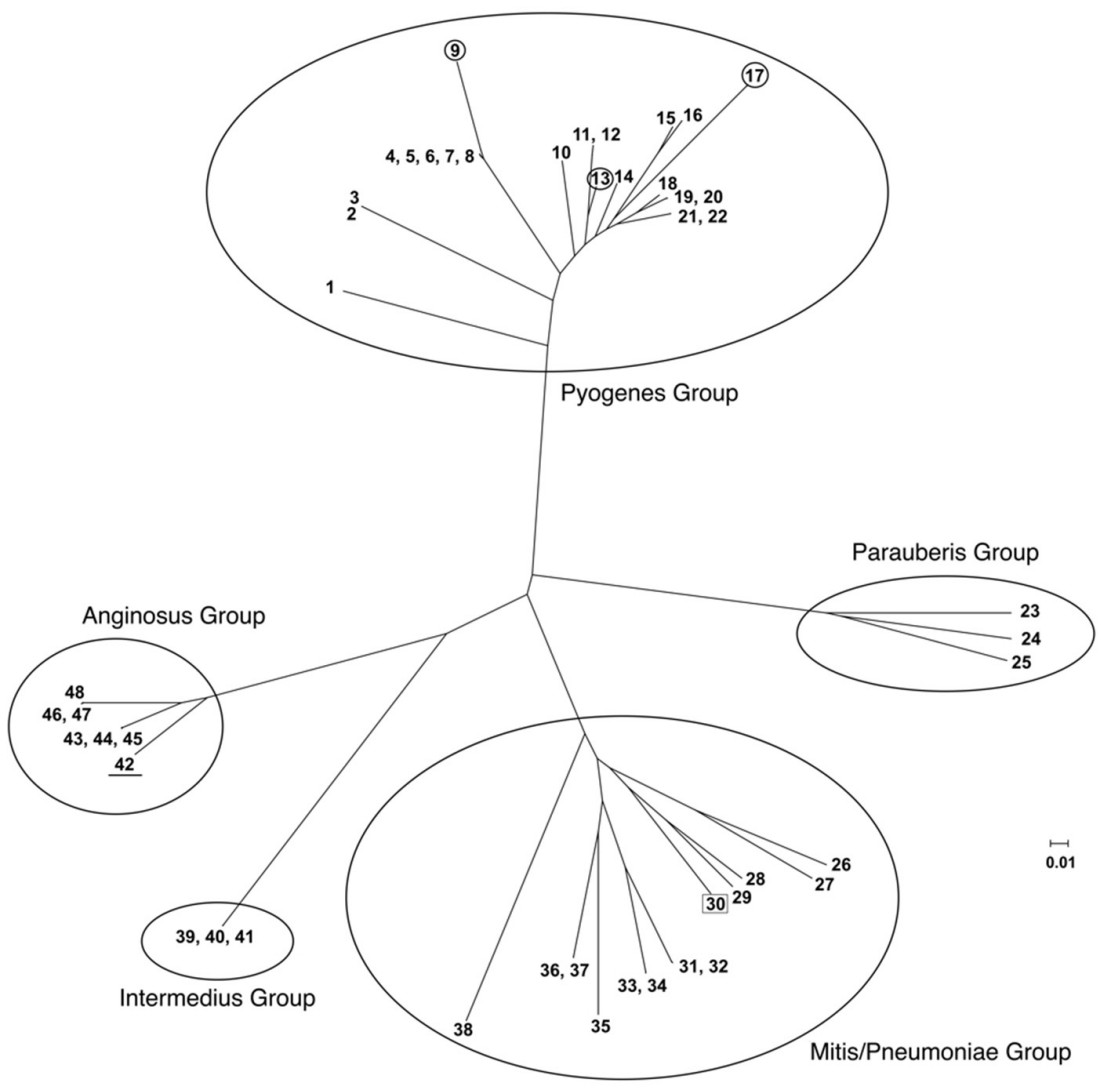
**Phylogenetic tree of streptococcal chromosomal islands integrated into DNA repair genes *mutL.*** Full genome trees are derived. In the Pyogenes group, all of which integrate into *mutL*, the circled members of the tree are CI found in the closely related species *S. dysgalactiae* subsp. *equisimilis* (SeqCI; nos. 9 and 13) and *S. canis* (ScaCI; no. 17). The SmiCI that integrates into *manA* in the Mitis/Pneumoniae group is boxed for identification; the remainder of the group integrates into *uvrA*. The members of the Anginosus and Intermedius groups integrate into *mutL* and have a conserved integrase gene; however, the remainder of their chromosomes is divergent, causing them to form separate phylogenetic groups. The one exception is that the *S. intermedius* strain F0413 SinCI (#42; underlined) is closely related to the SanCI and forms part of that group. The detailed legend for the individual members of the tree is presented in Supplemental Table 2.

Using the SpyCIM1 integrase gene as the query, a TBLASTN search (Altschul et al., 1997) of the available complete or partial bacterial genomes revealed many related islands in *Streptococcus* species that are integrated into the same attachment site in *mutL*, including *Streptococcus anginosus, Streptococcus intermedius, Streptococcus dysgalactiae* subsp. *equisimilus, Streptococcus canis*, and *Streptococcus parauberis* (Table 2). In these species, the CI may potentially regulate the MMR operon much like how SpyCIM1 does. Ignoring the defective SpyCIM5 integrase that has a 128 bp deletion in the gene (Scott et al., 2012), these integrases share at least 64.0% amino acid sequence identity (Supplemental Figure 1). Phylogenetic tree analysis of the integrases shows close similarity as well with little sequence distance (Figure 3). Perhaps this is not surprising, as these islands have conserved core sequences required for integration into streptococcal *mutL*, which itself provides a conserved target (Figure 3, insert). However, despite strong conservation of the integrase, other regions of these CI show considerable genetic diversity between species. Genomic alignment of the islands revealed four distinct groups within the MMR islands (Figure 4). The diversity and frequency of these islands is remarkable with chromosomal islands found in streptococcal species associated with human disease, streptococcosis in flounder and dairy bovine mastitis (Nho et al., 2011; Lefébure et al., 2012).

**Figure 5:**
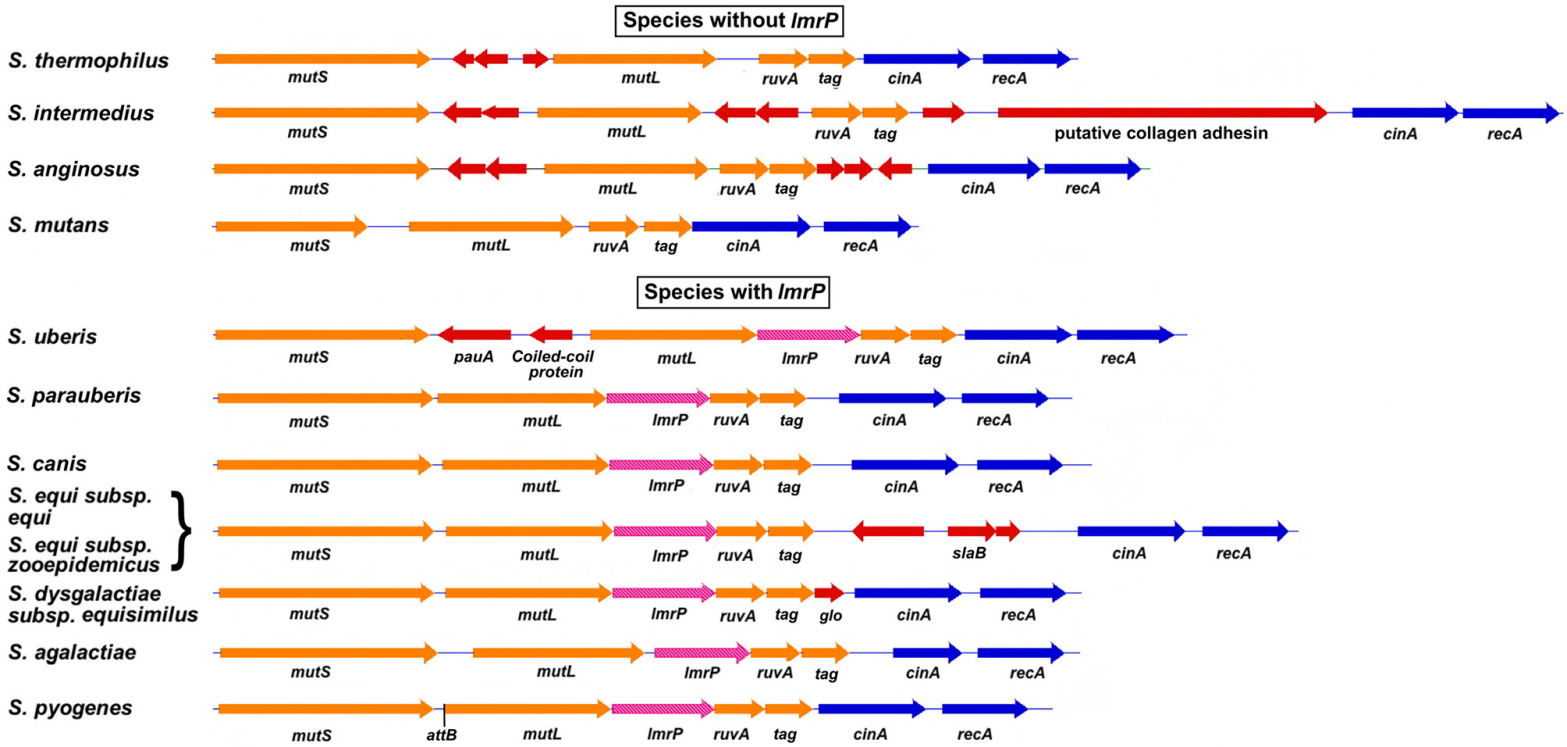
**The MMR operon region from selected streptococcal species**. The MMR operon in *S. pyogenes* and related streptococci is composed of a core group of genes involved in MMR, Holliday junction resolution and BER (*mutS, mutL, ruvA*, and *tag*). Indels are frequently found to supplement this basic genetic unit: MDR gene *lmrP* forms an additional part of this core in groups A, B, C, and G streptococci. Some indels encode proteins of unknown function, but others appear to encode potential virulence factors such as *pauA* (streptokinase) in *S. uberis* and the collagen-like adhesin in *S. intermedius*. For an extended list of streptococcal species that are differentiated by the presence or absence of *lmrP*, see Supplemental Table 1. The core genes of the streptococcal MMR operon are colored orange; MDP gene *lmrP* is magenta, and indels are red. The attachment site for SpyCI in *S. pyogenes* is indicated (*attB*). Legend for indel genes: *glo*—glyoxalase family protein; *slaB*—phospholipase A2 SlaB; *pauA*—streptokinase.

The MMR operon, as organized in *S. pyogenes*, is present in many other species of the genus *streptococcus*. However, there is one major differentiating characteristic in this operon that creates a division between these other species: the presence or absence of the gene for MDR LmrP (Figure 5 and Supplemental Table 1). Groups A, B, C, and G streptococci have *lmrP* inserted between *mutL* and *ruvA* as do a number of other species including *S. iniae, S. uberis*, and *S. parauberis*. While *S. mutans* and most viridans streptococci do not have *lmrP*, a few, like *S. oralis*, do. The presence or absence of this MDR gene, as well as its regulation by a chromosomal island, raises some interesting biological questions. Why is a gene for a drug efflux pump transcriptionally linked to DNA repair genes? Further, what is to be gained, if anything, by the cell through inhibition of expression of this gene? Indeed, why is this gene dispensable in some species but present in others? The answers to these questions will come as the role of this MDR protein in the biology and virulence of *S. pyogenes* and other species is determined. SpyCI-like CI that target *mutL* usually are found in species that have *lmrP*; however, *S. anginosus* and *S. intermedius*, both members of the milleri group that lack *lmrP*, have acquired CI that integrate into this gene.

In addition to CI identified by genome sequencing, another *S. anginosus* CI was identified from a clinical source. A daptomycin resistant strain of *S. anginosus* (strain J4206) was isolated from a patient with bacteremia and septic shock (Palacio et al., 2011) and was found to have a CI integrated into *mutL.* This CI (SanCI J4206) has been sequenced in our laboratory and its impact on the host mutator phenotype assessed (manuscript in preparation). Daptomycin resistance results from multistep genetic changes and is thought to be rare (Tran et al., 2013). As the related SpyCI are known to confer a mutator phenotype in their host, SanCI J4206 could putatively contribute to the emergence of daptomycin resistance in *S. anginosus* through hypermutability. Previously, daptomycin resistance has not observed in an *in vitro* study of 106 *S. anginosus* isolates (Streit et al., 2005).

It is possible that SpyCI, as well as other Gram-positive phage-like chromosomal islands (Novick et al., 2010), have a complex evolutionary history and their genetic material may have originated from disparate sources. While each chromosomal island shows considerable diversity (Scott et al., 2008), several genes, notably the integrase, primase, and replicase genes, are highly conserved, providing clues to the minimal genome composition needed for a functional CI.

## Streptococcal CI with other gene integration targets

**Figure 6:**
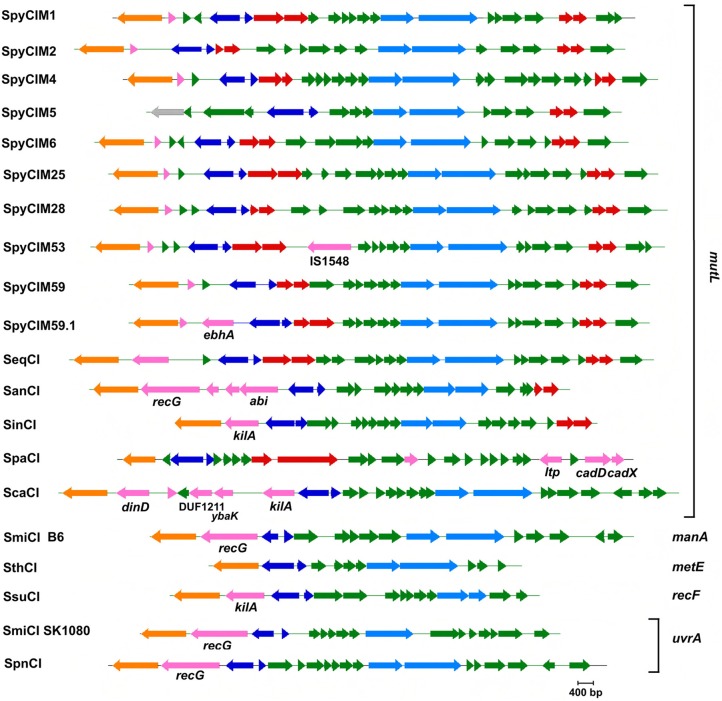
**The CI of *S. pyogenes* and related species**. The identification of each CI is shown to the left of its map, while the integration site (*attB*) is shown to the right of the map (see Tables 1, 2 for identification of CI and *attB* sites). INDELs with identifiable homologous genes are labeled by that homolog. Color key: orange—site-specific integrase; dark blue—control of lysogeny; light blue—DNA replication; red—maintenance; pink—INDELs; green—unknown function; gray—pseudogene. INDEL key: IS1548—insertion element 1548; *ebhA*—transmembrane surface adhesin; *recG*—recombination protein RecG superfamily; *abi*—abortive phage infection protein; *kilA*—plasmid maintenance protein; *ltp*—host cell surface-exposed lipoprotein; *cadD* and *cadX*—cadmium resistance proteins; *dinD*—DNA damage inducible protein; DUF1211—domain of unknown function; *ybaK*—prolyl tRNA synthetase. Maps were created using Gene Construction Kit (Textco BioSoftware, West Lebanon, NH).

Streptococcal chromosomal islands have been identified by genome sequencing that target genes other than *mutL* (Table 2 and Figure 6). Although the biological impact of integration into any of these genes is not yet known, some predictions may be made based upon whether the 5′ or 3′ end of the ORF is the point of site-specific recombination. For example, in three of the nine currently available genomes of *S. agalactiae*, a SagCI is found integrated into *rpsD*, which encodes the 30S ribosomal protein S4. Given the essential role of this protein in ribosome function, it is perhaps no surprise that this chromosomal island integrates into the 3′ end of the *rpsD* ORF so transcription is unimpeded. Other genes, by contrast, could be regulated in their expression patterns to the benefit of the cell, at least under certain environmental or physiological conditions. For example, the chromosomal island from the B6 strain of *Streptococcus mitis* (SmiCIB6) integrates into the 5′ end of the gene encoding alpha-1,2-mannosidase (*manA*) while the *Streptococcus thermophilus* CI (SthCI) integrates into *metE*, encoding methionine synthase. In both cases, a SpyCI-like switch could activate or silence these genes to optimize host fitness. Regulation of metE expression could alter the relative intracellular levels of homocysteine and methionine (Matthews et al., 1998), which under some conditions might be favorable to the cell. Similarly, *S. mitis* B6 may only need the action of alpha-1,2-mannosidase when a need arises to use glycan or glycoproteins as a carbon source as observed in other oral streptococci (Tarelli et al., 1998).

**Figure 7:**
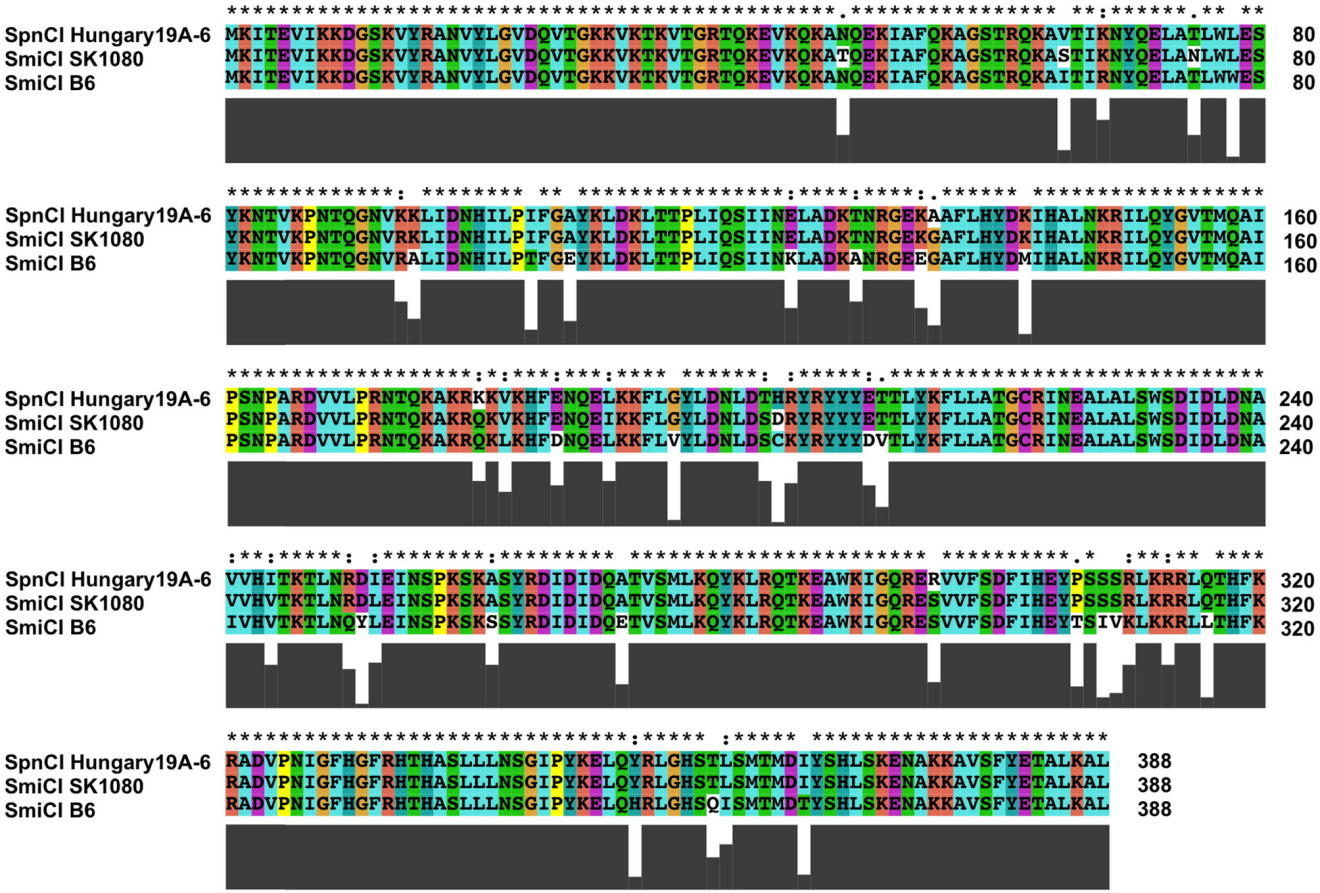
**Alignment of the SmiCI and SpnCI integrases that target either *manA* or *uvrA***. The integrase proteins encoded by the *S. mitis* SmiCI B6, the *S. mitis* SmiCI SK1080, and *S. pneumoniae* Hungary19A-6 SpnCI were aligned using ClustalX (Jeanmougin et al., 1998). The integrases from SpnCI Hungary19A-6 and SmiCI SK1080 both recognize an identical attachment site in the promoter of *uvrA*, while the SmiCI B6 integrase recognizes *manA*. Above the alignment, an asterisk (^*^) indicates identity between the three proteins, a colon (:) indicates a strongly conservative amino acid substitution, and a dot (.) indicates a weakly conservative amino acid substitution

The *S. mitis* SmiCI provide an interesting glimpse into the evolution and diversification of phage-like CI. As discussed above, SmiCI B6 integrates into *manA*. However, the related SmiCI from other *S. mitis* strains as well as the SpnCI from *S. pneumoniae* target the probable operator controlling the expression of the gene (*uvrA*) encoding the A subunit of the UvrABC excinuclease that is a key component of nucleotide excision repair (NER). Remarkably, the SmiCI B6 integrase has 98% homology at the protein level with the other SmiCI and the SpnCI integrases (Figure 7), even though they target separate DNA genes (*manA* and *uvrA*, respectively). This observation suggests that only a relatively few amino acid changes were necessary to expand the gene repertoire of this CI, creating new possibilities for altering host expression patterns. The SmiCI and SpnCI targeting of *uvrA*, a key gene for another essential DNA repair pathway (NER), suggests that ability to selectively adopt a mutator phenotype in a regulated fashion provides a selectable advantage for these cells. Based upon the example of SpyCI in *S. pyogenes*, one could predict that the SpnCI and SmiCI will have a cycle of excision and re-integration in response to the growth state of the cell or some environmental sensor.

## Indels and CI-mediated phenotypes

Many members of the streptococcal phage-like CI have notable indels (insertions and deletions) in their genomes that may contribute to the host phenotype (Figure 6). We previously reported on the impact of the 128 bp deletion in the SpyCIM5 integrase gene on *S. pyogenes* strain Manfredo and the solution the cell adopted to prevent permanent silencing of *mutL* and the downstream genes of the operon (Scott et al., 2012). The SpyCIM53 element from strain Alab49 (Bessen et al., 2011) is genetically quite related to SpyCiM1 from strain SF370 but has acquired the insertion element IS1548 (Figures 4, 6). The recently reported severe disease associated, epidemic strain MGAS15252 (Fittipaldi et al., 2012) contains SpyCIM59.1, a CI with an indel (*ebhA*) predicted to encode extracellular matrix-binding protein with a transmembrane domain, which could be a potential virulence factor.

The phage-like CI from the non-*pyogenes* streptococcal species often contain indels that may contribute to host fitness or prevent elimination of the CI. A gene encoding an ATPase related to the RecG superfamily family of proteins is found in the *S. anginosus* SanCI as well as the phylogenetically separate SmiCI from *S. mitis* and SpnCI from *S. pneumoniae* (Figures 4, 6). Protein RecG provides a means of rescuing stalled replication forks in *E. coli* (Briggs et al., 2004), and these CI-encoded homologs might offset some aspects of the mutator phenotype caused by the integration of the CI into *mutL* (SanCI) or *uvrA* (SmiCI and SpnCI). Another CI indel that frequently appears is a homolog of the KilA family of proteins (found in the SinCI, ScaCI, and SsuCI genomes). The *kilA* gene was identified in the broad-host-range *E. coli* plasmid RK2 and originally detected by the potential lethality of its gene product to the host cell (Goncharoff et al., 1991). Based upon previously reported criteria (Makarova et al., 2009), a number of potential toxin-antitoxin genes are found in the streptococcal phage-like CI that may function to prevent elimination of the CI from the host during replication and cell division (Figure 6), and these KilA homologs may also contribute to CI maintenance.

Other streptococcal phage-like CI indels may improve host fitness to environmental challenges. Resistance to cadmium and other heavy metals in microbes occurs frequently, which interestingly is often co-selected with antibiotic resistance and may contribute to the persistence of resistance genes in the environment (Baker-Austin et al., 2006). The *S. parauberis* SpaCI has acquired the *cadDX* cadmium resistance operon that is related to ones found in *S. aureus* and *Streptococcus salivarius* (O'Brien et al., 2002; Chen et al., 2008). Many heavy metal resistance mechanisms are able to act upon multiple substrates (Baker-Austin et al., 2006), and the principal substrate for resistance conferred by this SpaCI operon in *S. parauberis* is unknown. However, since cadD encodes a transmembrane protein, its gene product will alter the surface properties of the host cell, which could not only confer resistance but also alter antigenicity and charge of the cell membrane. The SpaCI also encodes a second protein with the potential for altering the surface of its host: Ltp, a member of a family of phage encoded lipoproteins that are involved in superinfection exclusion. Proteins of this family have been shown to act at the stage of DNA release from the phage head into the cell (Neve et al., 1998), thus interfering with the lytic cycle of phages that might infect *S. parauberis*. Other streptococcal phage-like CI also carry genes that may protect their host cell from infection by invading lytic phages or induction of endogenous prophages to the lytic cycle. The SanCI of *S. anginosus* encode the gene *abi*, which encodes a protein that is a member of a protein family that mediates bacteriophage resistance by causing abortive infection in *Lactococcus* species (Anba et al., 1995; Bidnenko et al., 1995). The SanCI encoded Abi protein has a predicted helix-turn-helix DNA binding motif, which in these proteins is thought to play a role in the interference of the phage life cycle through altering the transcriptional program of the virus. In addition to the *kilA* gene discussed above, the *S. canis* ScaCI contains several other indels that may promote host fitness through protection of cell metabolism. Located immediately upstream of *int* is a gene encoding a homolog of the DinD DNA damage induced protein. In *E. coli*, the DinD protein inhibits RecA-mediated DNA strand exchange, which may limit unwanted homologous recombination (Uranga et al., 2011). Another ScaCI gene (*ybaK*) also may encode a system to increase host cell fitness. Members of the YbaK protein family have deacylase domains are trans-acting amino acid-editing class II prolyl tRNA synthetases, whose primary function is to hydrolyze mischarged cysteinyl-tRNA^Pro^, thus ensuring the fidelity of translation and prevent accumulation of mistranslated proteins (Kumar et al., 2012; Das et al., 2014).

## CI as regulatory elements of the host phenotype

While the regulation of MMR by SpyCI in *S. pyogenes* and other streptococci is a remarkable evolutionary adaptation, it is one of a number of examples of how phages and phage-like mobile genetic elements have evolved to form a beneficial relationship with their host bacterium. In *E. coli*, several examples of integrated phage-like elements affecting gene sequence or expression have been reported. In strain K-12, a P-4-like cryptic prophage controls expression of the AlpA transcriptional regulator by site-specific recombination. Overexpression of *alpA* leads to suppression of capsule overproduction and UV sensitivity in cells defective for the Lon protease (Kirby et al., 1994; Trempy et al., 1994). The integration of the cryptic prophage suppresses *alpA* expression and restores normal capsule production and UV sensitivity. Unlike the dynamic cycle of MMR control by SpyCIM1, excision of the cryptic prophage leads to the elements loss and a permanent conversion of cell phenotype (Kirby et al., 1994). Similarly, integration of phage lambda into a secondary attachment site at the *guaB* promoter inhibits the expression of inosine monophosphate (IMP) dehydrogenase (Thomas and Drabble, 1986). In this case, though, the selective benefit of the resulting inhibition of *de novo* purine biosynthesis following integration is uncertain and may be just an unfavorable event that would be unstable over time. The integration of Lambdoid phage 21 replaces 165 bp of the 3′ end of the isocitrate dehydrogenase gene *icd* to provide an alternative ending (Campbell et al., 1992), which again is an event of unknown biological impact that may warrant further study.

A number of Gram-positive examples of gene control by mobile phage-like elements have also been reported. The sigma K intervening element (*skin*) of *Bacillus subtilis* is a 48 kB prophage-like element that integrates into *sigK*, separating the gene into regions historically known as *spoIIIC* and *spoIVCB* (Kunkel et al., 1990). Excision of the *skin* element from the chromosome leads to reconstitution of the *sigK* gene, which during sporulation encodes the mother-cell-specific s^K^ factor (Stragier et al., 1989). Likewise, a smaller, 14.6 kb element (*skin^Cd^*) was found to interrupt *sigK* in *Clostridium difficile*; unlike in *B. subtilis* where *skin* may be deleted without major impact upon sporulation, the *C. difficile skin^Cd^* is required for efficient completion of this event (Haraldsen and Sonenshein, 2003). Interestingly, phylogenetic analysis shows that these *skin* elements arose independently in *Bacillus* and *Clostridium*, leading to the speculation that this unusual form of *sigK* regulation may have some specific selective advantage in the regulation of sporulation. In *Listeria monocytogenes*, a recent report showed that the DNA uptake competence system, considered non-functional, has a temperate prophage integrated into *comK* (Rabinovich et al., 2012). Surprisingly, the *L. monocytogenes* Com system promoted bacterial escape from macrophages, and the regulation of this system depended upon the activation of *comK* following prophage excision, which was specifically induced during intracellular growth, reminiscent of the activation of SpyCIM1 activation at the onset of exponential phase (Scott et al., 2008). Recently, a phage-like chromosomal island from the genome of *Enterococcus faecalis* V583 has been described and its requirement for a helper phage for packaging demonstrated (Matos et al., 2013). The integration target site for this element is identified as the promoter of a xanthine/uracil permeases family of genes; however, the precise function of this gene in *E. faecalis* or the impact of the chromosomal island integration into this gene remains unknown.

The relation between mobile genetic elements and host gene expression is a key component of the biology of *S. aureus*, which in many ways resembles these relationships in *S. pyogenes*. These elements range from typical lambdoid prophages to the SaPI phage-like chromosomal islands. *S. aureus* prophages have been demonstrated to mediate gene conversion by controlling the expression of lipase, β-Lysin, Staphylokinase, and Enterotoxin A (Lee and Iandolo, 1985; Coleman et al., 1989; Zabicka et al., 1993), while the SaPI are vectors for the toxic shock syndrome toxin (TSST) (Lindsay et al., 1998; Novick et al., 2010). In addition to being vectors for TSST, the SaPI carry other genes that modify the host phenotype such as a biofilm-associated protein (BAP) (Ubeda et al., 2003) or von Willebrand factor-binding protein (Lindsay et al., 1998; Viana et al., 2010).

In their pioneering paper demonstrating the unexpectedly high frequency of the mutator phenotype in wild populations of bacteria, LeClerc and co-workers observed that “the ultimate pathogen would possess an elevated mutation rate that is transient (or conditional), providing genetic variation during the first few hours when the pathogen must survive, invade, and colonize its host” (LeClerc et al., 1996). The SpyCI of *S. pyogenes* and the similar CI that colonize related species of the genus streptococcus may well be an example of a system that fulfills this prediction by their unique mechanism of MMR control. The frequent occurrence of MMR defects in natural bacterial populations argues that a selective benefit exists in this phenotype, whether it stems from increased mutability or the potential for horizontal gene transfer (Matic et al., 1995). In most species of bacteria, however, the mutator phenotype is fixed and poses a distinct risk to the cell in the form of acquiring unwanted mutations that might lead to decreased viability. So far, only in the streptococci has a system been discovered that allows the cell to switch between a mutator and wild type phenotype to presumably achieve a balance between costs and benefits. The presence of other phage-like CI in the various streptococcal species that potentially target other genes for regulation suggests that these elements may be an important aspect of the biology of these low G+C% bacteria. The widespread occurrence by SpyCI and related CI in the pathogenic streptococci may be a clue to their importance to the virulence and survival of these bacteria, which may prove ultimately to be as significant as the carriage of toxigenic bacteriophages.

### Conflict of interest statement

The authors declare that the research was conducted in the absence of any commercial or financial relationships that could be construed as a potential conflict of interest.

## Supplementary material

Supplemental Figure S1 **Identity Matrix of Integrases**. An amino acid sequence identity matrix of the CI integrase proteins from *streptococcus* species that target *mutL* is presented. SpyCI integrases show a high degree of similarity with each other, as well as strong similarities to ones found in *S. canis* and *S. dysgalactiae* subspecies *equisimilis*. High amino acid similarities suggest that core sequences are conserved and required for integration into *mutL*.

## References

[B1] Altschul S. F. Madden T. L. Schaffer A. A. Zhang J. Zhang Z. Miller W. (1997). Gapped BLAST and PSI-BLAST: a new generation of protein database search programs. Nucleic Acids Res. 25, 3389–3402 10.1093/nar/25.17.3389 9254694 PMC146917

[B2] Anba J. Bidnenko E. Hillier A. Ehrlich D. Chopin M. C. (1995). Characterization of the lactococcal abiD1 gene coding for phage abortive infection. J. Bacteriol. 177, 3818–3823 7601848 10.1128/jb.177.13.3818-3823.1995 PMC177101

[B3] Baker-Austin C. Wright M. S. Stepanauskas R. McArthur J. V. (2006). Co-selection of antibiotic and metal resistance. Trends Microbiol. 14, 176–182 10.1016/j.tim.2006.02.006 16537105

[B4] Banks D. J. Beres S. B. Musser J. M. (2002). The fundamental contribution of phages to GAS evolution, genome diversification and strain emergence. Trends Microbiol. 10, 515–521 10.1016/S0966-842X(02)02461-7 12419616

[B5] Bayliss C. D. Sweetman W. A. Moxon E. R. (2004). Mutations in *Haemophilus influenzae* mismatch repair genes increase mutation rates of dinucleotide repeat tracts but not dinucleotide repeat-driven pilin phase variation rates. J. Bacteriol. 186, 2928–2935 10.1128/JB.186.10.2928-2935.2004 15126452 PMC400628

[B6] Bessen D. E. Kumar N. Hall G. S. Riley D. R. Luo F. Lizano S. (2011). Whole-genome association study on tissue tropism phenotypes in group A Streptococcus. J. Bacteriol. 193, 6651–6663 10.1128/JB.05263-11 21949075 PMC3232895

[B7] Bidnenko E. Ehrlich D. Chopin M. C. (1995). Phage operon involved in sensitivity to the *Lactococcus lactis* abortive infection mechanism AbiD1. J. Bacteriol. 177, 3824–3829 7601849 10.1128/jb.177.13.3824-3829.1995 PMC177102

[B8] Bjelland S. Bjoras M. Seeberg E. (1993). Excision of 3-methylguanine from alkylated DNA by 3-methyladenine DNA glycosylase I of *Escherichia coli*. Nucleic Acids Res. 21, 2045–2049 10.1093/nar/21.9.2045 8502545 PMC309463

[B9] Bjorkholm B. Sjolund M. Falk P. G. Berg O. G. Engstrand L. Andersson D. I. (2001). Mutation frequency and biological cost of antibiotic resistance in *Helicobacter pylori*. Proc. Natl. Acad. Sci. U.S.A. 98, 14607–14612 10.1073/pnas.241517298 11717398 PMC64729

[B10] Bolhuis H. Poelarends G. van Veen H. W. Poolman B. Driessen A. J. Konings W. N. (1995). The Lactococcal lmrP gene encodes a proton motive force-dependent drug transporter. J. Biol. Chem. 270, 26092–26098 10.1074/jbc.270.44.26092 7592810

[B11] Briggs G. S. Mahdi A. A. Weller G. R. Wen Q. Lloyd R. G. (2004). Interplay between DNA replication, recombination and repair based on the structure of RecG helicase. Philos. Trans. R. Soc. Lond. B Biol. Sci. 359, 49–59 10.1098/rstb.2003.1364 15065656 PMC1693295

[B12] Brussow H. Canchaya C. Hardt W. D. (2004). Phages and the evolution of bacterial pathogens: from genomic rearrangements to lysogenic conversion. Microbiol. Mol. Biol. Rev. 68, 560–602 10.1128/MMBR.68.3.560-602.2004 15353570 PMC515249

[B13] Campbell A. Schneider S. J. Song B. (1992). Lambdoid phages as elements of bacterial genomes. Genetica 86, 259–267 10.1007/BF00133724 1468648

[B14] Canchaya C. Desiere F. McShan W. M. Ferretti J. J. Parkhill J. Brussow H. (2002). Genome analysis of an inducible prophage and prophage remnants integrated in the *Streptococcus pyogenes* strain SF370. Virology 302, 245–258 10.1006/viro.2002.1570 12441069

[B15] Carapetis J. R. Steer A. C. Mulholland E. K. Weber M. (2005). The global burden of group A streptococcal diseases. Lancet Infect. Dis. 5, 685–694 10.1016/S1473-3099(05)70267-X 16253886

[B16] Chen Y. Y. Feng C. W. Chiu C. F. Burne R. A. (2008). cadDX operon of *Streptococcus salivarius* 57.I. Appl. Environ. Microbiol. 74, 1642–1645 10.1128/AEM.01878-07 18165364 PMC2258612

[B17] Coleman D. Knights J. Russell R. Shanley D. Birkbeck T. H. Dougan G. (1991). Insertional inactivation of the *Staphylococcus aureus* beta-toxin by bacteriophage f13 occurs by site- and orientation-specific integration of the f13 genome. Mol. Microbiol. 5, 933–939 10.1111/j.1365-2958.1991.tb00768.x 1830359

[B18] Coleman D. C. Sullivan D. J. Russell R. J. Arbuthnott J. P. Carey B. F. Pomeray H. M. (1989). *Staphylococcus aureus* bacteriophages mediating the simultaneous lysogenic conversion of beta-lysin, staphylokinase and enterotoxin A: molecular mechanism of triple conversion. J. Gen. Microbiol. 135, 1679–1697 2533245 10.1099/00221287-135-6-1679

[B19] Crimmins G. T. Herskovits A. A. Rehder K. Sivick K. E. Lauer P. Dubensky T. W. Jr. (2008). *Listeria monocytogenes* multidrug resistance transporters activate a cytosolic surveillance pathway of innate immunity. Proc. Natl. Acad. Sci. U.S.A. 105, 10191–10196 10.1073/pnas.0804170105 18632558 PMC2481368

[B20] Crooks G. E. Hon G. Chandonia J. M. Brenner S. E. (2004). WebLogo: a sequence logo generator. Genome Res. 14, 1188–1190 10.1101/gr.849004 15173120 PMC419797

[B21] Das M. Vargas-Rodriguez O. Goto Y. Suga H. Musier-Forsyth K. (2014). Distinct tRNA recognition strategies used by a homologous family of editing domains prevent mistranslation. Nucleic Acids Res. 42, 3943–3953 10.1093/nar/gkt1332 24371276 PMC3973320

[B22] Desiere F. McShan W. M. van Sinderen D. Ferretti J. J. Brussow H. (2001). Comparative genomics reveals close genetic relationships between phages from dairy bacteria and pathogenic Streptococci: evolutionary implications for prophage-host interactions. Virology 288, 325–341 10.1006/viro.2001.1085 11601904

[B23] Drummond A. Ashton B. Buxton S. Cheung M. Cooper A. Heled J. (2012). Geneious v6.1.7. Available online at: http://www.geneious.com

[B25] Ferretti J. J. McShan W. M. Ajdic D. Savic D. J. Savic G. Lyon K. (2001). Complete genome sequence of an M1 strain of *Streptococcus pyogenes*. Proc. Natl. Acad. Sci. U.S.A. 98, 4658–4663 10.1073/pnas.071559398 11296296 PMC31890

[B26] Fittipaldi N. Beres S. B. Olsen R. J. Kapur V. Shea P. R. Watkins M. E. (2012). Full-Genome dissection of an epidemic of severe invasive disease caused by a hypervirulent, recently emerged clone of group A *Streptococcus*. Am. J. Pathol. 180, 1522–1534 10.1016/j.ajpath.2011.12.037 22330677

[B27] Fouts D. E. (2006). Phage_Finder: automated identification and classification of prophage regions in complete bacterial genome sequences. Nucleic Acids Res. 34, 5839–5851 10.1093/nar/gkl732 17062630 PMC1635311

[B28] Geis A. El Demerdash H. A. Heller K. J. (2003). Sequence analysis and characterization of plasmids from *Streptococcus thermophilus*. Plasmid 50, 53–69 10.1016/S0147-619X(03)00029-5 12826058

[B29] Goncharoff P. Saadi S. Chang C. H. Saltman L. H. Figurski D. H. (1991). Structural, molecular, and genetic analysis of the *kilA* operon of broad-host-range plasmid RK2. J. Bacteriol. 173, 3463–3477 2045366 10.1128/jb.173.11.3463-3477.1991 PMC207960

[B30] Haraldsen J. D. Sonenshein A. L. (2003). Efficient sporulation in *Clostridium difficile* requires disruption of the sigmaK gene. Mol. Microbiol. 48, 811–821 10.1046/j.1365-2958.2003.03471.x 12694623

[B31] Iwasaki H. Shiba T. Nakata A. Shinagawa H. (1989). Involvement in DNA repair of the *ruvA* gene of *Escherichia coli*. Mol. Gen. Genet. 219, 328–331 10.1007/BF00261196 2693946

[B32] Jeanmougin F. Thompson J. D. Gouy M. Higgins D. G. Gibson T. J. (1998). Multiple sequence alignment with Clustal X. Trends Biochem. Sci. 23, 403–405 10.1016/S0968-0004(98)01285-7 9810230

[B33] Kaasen I. Evensen G. Seeberg E. (1986). Amplified expression of the *tag* + and *alkA* + genes in *Escherichia coli*: identification of gene products and effects on alkylation resistance. J. Bacteriol. 168, 642–647 3536857 10.1128/jb.168.2.642-647.1986 PMC213529

[B34] Kaplan D. L. O'Donnell M. (2006). RuvA is a sliding collar that protects Holliday junctions from unwinding while promoting branch migration. J. Mol. Biol. 355, 473–490 10.1016/j.jmb.2005.10.075 16324713

[B35] Kataja J. Huovinen P. Skurnik M. Seppala H. (1999). Erythromycin resistance genes in group A streptococci in Finland. The finnish study group for antimicrobial resistance. Antimicrob. Agents Chemother. 43, 48–52 9869564 10.1128/aac.43.1.48 PMC89019

[B36] Kirby J. E. Trempy J. E. Gottesman S. (1994). Excision of a P4-like cryptic prophage leads to Alp protease expression in *Escherichia coli*. J. Bacteriol. 176, 2068–2081 7511583 10.1128/jb.176.7.2068-2081.1994 PMC205313

[B37] Kumar S. Das M. Hadad C. M. Musier-Forsyth K. (2012). Substrate and enzyme functional groups contribute to translational quality control by bacterial prolyl-tRNA synthetase. J. Phys. Chem. B 116, 6991–6999 10.1021/jp300845h 22458656 PMC3376218

[B38] Kunkel B. Losick R. Stragier P. (1990). The *Bacillus subtilis* gene for the development transcription factor sigma K is generated by excision of a dispensable DNA element containing a sporulation recombinase gene. Genes Dev. 4, 525–535 10.1101/gad.4.4.525 2163341

[B39] LeClerc J. E. Cebula T. A. (2000). *Pseudomonas* survival strategies in cystic fibrosis. Science 289, 391–392 10.1126/science.289.5478.391c 10939947

[B40] LeClerc J. E. Li B. Payne W. L. Cebula T. A. (1996). High mutation frequencies among *Escherichia coli* and *Salmonella* pathogens. Science 274, 1208–1211 10.1126/science.274.5290.1208 8895473

[B41] Lee C. Y. Iandolo J. J. (1985). Mechanism of bacteriophage conversion of lipase activity in *Staphylococcus aureus*. J. Bacteriol. 164, 288–293 2995312 10.1128/jb.164.1.288-293.1985 PMC214242

[B42] Lee C. Y. Iandolo J. J. (1986). Lysogenic conversion of staphylococcal lipase is caused by insertion of the bacteriophage L54a genome into the lipase structural gene. J. Bacteriol. 166, 385–391 3009394 10.1128/jb.166.2.385-391.1986 PMC214616

[B43] Lefébure T. Richards V. P. Lang P. Pavinski-Bitar P. Stanhope M. J. (2012). Gene repertoire evolution of *Streptococcus pyogenes* inferred from Phylogenomic analysis with *Streptococcus canis* and *Streptococcus dysgalactiae*. PLoS ONE 7:e37607 10.1371/journal.pone.0037607 22666370 PMC3364286

[B44] Li G. M. (2008). Mechanisms and functions of DNA mismatch repair. Cell Res. 18, 85–98 10.1038/cr.2007.115 18157157

[B45] Limia A. Jimenez M. L. Delgado T. Sanchez I. Lopez S. Lopez-Brea M. (1998). [Phenotypic characterization of erythromycin resistance in strains of the genus Streptococcus isolated from clinical specimens]. Rev. Esp. Quimioter. 11, 216–220 9795307

[B46] Lindsay J. A. Ruzin A. Ross H. F. Kurepina N. Novick R. P. (1998). The gene for toxic shock toxin is carried by a family of mobile pathogenicity islands in *Staphylococcus aureus*. Mol. Microbiol. 29, 527–543 10.1046/j.1365-2958.1998.00947.x 9720870

[B47] Louie A. Brown D. L. Liu W. Kulawy R. W. Deziel M. R. Drusano G. L. (2007). *In vitro* infection model characterizing the effect of efflux pump inhibition on prevention of resistance to levofloxacin and ciprofloxacin in *Streptococcus pneumoniae*. Antimicrob. Agents Chemother. 51, 3988–4000 10.1128/AAC.00391-07 17846144 PMC2151412

[B48] Makarova K. S. Wolf Y. I. Koonin E. V. (2009). Comprehensive comparative-genomic analysis of type 2 toxin-antitoxin systems and related mobile stress response systems in prokaryotes. Biol. Direct 4:19 10.1186/1745-6150-4-19 19493340 PMC2701414

[B49] Mason R. E. Allen W. E. (1975). Characteristics of *Staphylococcus aureus* associated with lysogenic conversion to loss of beta-hemolysin production. Can. J. Microbiol. 21, 1113–1116 10.1139/m75-161 125146

[B50] Matic I. Radman M. Taddei F. Picard B. Doit C. Bingen E. (1997). Highly variable mutation rates in commensal and pathogenic *Escherichia coli*. Science 277, 1833–1834 10.1126/science.277.5333.1833 9324769

[B51] Matic I. Rayssiguier C. Radman M. (1995). Interspecies gene exchange in bacteria: the role of SOS and mismatch repair systems in evolution of species. Cell 80, 507–515 10.1016/0092-8674(95)90501-4 7859291

[B52] Matic I. Taddei F. Radman M. (2000). No genetic barriers between *Salmonella enterica* serovar typhimurium and *Escherichia coli* in SOS-induced mismatch repair-deficient cells. J. Bacteriol. 182, 5922–5924 10.1128/JB.182.20.5922-5924.2000 11004198 PMC94721

[B53] Matos R. Lapaque N. Rigottier-Gois L. Debarbieux L. Meylheuc T. Gonzalez-Zorn B. (2013). *Enterococcus faecalis* prophage dynamics and contributions to pathogenic traits. PLoS Genet. 9:e1003539 10.1371/journal.pgen.1003539 23754962 PMC3675006

[B54] Matthews R. G. Sheppard C. Goulding C. (1998). Methylenetetrahydrofolate reductase and methionine synthase: biochemistry and molecular biology. Eur. J. Pediatr. 157 Suppl. 2, S54–S59 10.1007/PL00014305 9587027

[B55] McShan W. M. (2005). The bacteriophages of group A Streptococci, in Gram-Positive Pathogens, 2nd Edn., eds Fischetti V. A. Novick R. P. Ferretti J. J. Portnoy D. A. Rood J. I. (Washington, DC: American Society for Microbiology), 123–142

[B56] McShan W. M. Ferretti J. J. (2007). Bacteriophages and the host phenotype, in Bacteriophages: Genetics and Molecular Biology, ed McGrath S. (Norwich: Horizon Scientific Press), 229–250

[B57] McShan W. M. Ferretti J. J. Karasawa T. Suvorov A. N. Lin S. Qin B. (2008). Genome sequence of a nephritogenic and highly transformable M49 strain of *Streptococcus pyogenes*. J. Bacteriol. 190, 7773–7785 10.1128/JB.00672-08 18820018 PMC2583620

[B58] Neve H. Zenz K. I. Desiere F. Koch A. Heller K. J. Brussow H. (1998). Comparison of the lysogeny modules from the temperate *Streptococcus thermophilus* bacteriophages TP-J34 and Sfi21: implications for the modular theory of phage evolution. Virology 241, 61–72 10.1006/viro.1997.8960 9454717

[B59] Nho S. W. Hikima J. Cha I. S. Park S. B. Jang H. B. del Castillo C. S. (2011). Complete genome sequence and immunoproteomic analyses of the bacterial fish pathogen *Streptococcus parauberis*. J. Bacteriol. 193, 3356–3366 10.1128/JB.00182-11 21531805 PMC3133276

[B60] Novick R. P. Christie G. E. Penades J. R. (2010). The phage-related chromosomal islands of Gram-positive bacteria. Nat. Rev. Microbiol. 8, 541–551 10.1038/nrmicro2393 20634809 PMC3522866

[B61] O'Brien F. G. Price C. Grubb W. B. Gustafson J. E. (2002). Genetic characterization of the fusidic acid and cadmium resistance determinants of *Staphylococcus aureus* plasmid pUB101. J. Antimicrob. Chemother. 50, 313–321 10.1093/jac/dkf153 12205055

[B62] Oliver A. Baquero F. Blazquez J. (2002). The mismatch repair system (*mutS, mutL* and *uvrD* genes) in *Pseudomonas aeruginosa*: molecular characterization of naturally occurring mutants. Mol. Microbiol. 43, 1641–1650 10.1046/j.1365-2958.2002.02855.x 11952911

[B63] Palacio F. Lewis J. S. 2nd Sadkowski L. Echevarria K. Jorgensen J. H. (2011). Breakthrough bacteremia and septic shock due to *Streptococcus anginosus* resistant to daptomycin in a patient receiving daptomycin therapy. Antimicrob. Agents Chemother. 55, 3639–3640 10.1128/AAC.00231-11 21502623 PMC3122392

[B64] Prunier A. L. Leclercq R. (2005). Role of *mutS* and *mutL* genes in hypermutability and recombination in *Staphylococcus aureus*. J. Bacteriol. 187, 3455–3464 10.1128/JB.187.10.3455-3464.2005 15866932 PMC1112015

[B65] Putman M. van Veen H. W. Degener J. E. Konings W. N. (2001). The lactococcal secondary multidrug transporter LmrP confers resistance to lincosamides, macrolides, streptogramins and tetracyclines. Microbiology 147, 2873–2880 11577166 10.1099/00221287-147-10-2873

[B66] Rabinovich L. Sigal N. Borovok I. Nir-Paz R. Herskovits A. A. (2012). Prophage excision activates *Listeria* competence genes that promote phagosomal escape and virulence. Cell 150, 792–802 10.1016/j.cell.2012.06.036 22901809

[B67] Richardson A. R. Yu Z. Popovic T. Stojiljkovic I. (2002). Mutator clones of *Neisseria meningitidis* in epidemic serogroup A disease. Proc. Natl. Acad. Sci. U.S.A. 99, 6103–6107 10.1073/pnas.092568699 11983903 PMC122909

[B68] Scott J. Nguyen S. King C. J. Hendrickson C. McShan W. M. (2012). Mutator phenotype prophages in the genome strains of *Streptococcus pyogenes*: control by growth state and by a cryptic prophage-encoded promoter. Front. Microbiol. 3:317 10.3389/fmicb.2012.00317 22969756 PMC3430984

[B69] Scott J. Thompson-Mayberry P. Lahmamsi S. King C. J. McShan W. M. (2008). Phage-associated mutator phenotype in group A *Streptococcus*. J. Bacteriol. 190, 6290–6301 10.1128/JB.01569-07 18676670 PMC2565987

[B70] Stragier P. Kunkel B. Kroos L. Losick R. (1989). Chromosomal rearrangement generating a composite gene for a developmental transcription factor. Science 243, 507–512 10.1126/science.2536191 2536191

[B71] Streit J. M. Steenbergen J. N. Thorne G. M. Alder J. Jones R. N. (2005). Daptomycin tested against 915 bloodstream isolates of viridans group streptococci (eight species) and *Streptococcus bovis*. J. Antimicrob. Chemother. 55, 574–578 10.1093/jac/dki032 15722390

[B72] Suvorov A. N. Polyakova E. M. McShan W. M. Ferretti J. J. (2009). Bacteriophage content of M49 strains of *Streptococcus pyogenes*. FEMS Microbiol. Lett. 294, 9–15 10.1111/j.1574-6968.2009.01538.x 19493003

[B73] Tarelli E. Byers H. L. Homer K. A. Beighton D. (1998). Evidence for mannosidase activities in *Streptococcus oralis* when grown on glycoproteins as carbohydrate source. Carbohydr. Res. 312, 159–164 10.1016/S0008-6215(98)00246-8 9836455

[B74] Thomas M. S. Drabble W. T. (1986). Secondary attachment site for bacteriophage lambda in the *gua*B gene of *Escherichia coli*. J. Bacteriol. 168, 1048–1050 2877966 10.1128/jb.168.2.1048-1050.1986 PMC213595

[B75] Tran T. T. Panesso D. Gao H. Roh J. H. Munita J. M. Reyes J. (2013). Whole-genome analysis of a daptomycin-susceptible *Enterococcus faecium* strain and its daptomycin-resistant variant arising during therapy. Antimicrob. Agents Chemother. 57, 261–268 10.1128/AAC.01454-12 23114757 PMC3535923

[B76] Trempy J. E. Kirby J. E. Gottesman S. (1994). Alp suppression of Lon: dependence on the *slpA* gene. J. Bacteriol. 176, 2061–2067 7511582 10.1128/jb.176.7.2061-2067.1994 PMC205312

[B77] Trong H. N. Prunier A. L. Leclercq R. (2005). Hypermutable and fluoroquinolone-resistant clinical isolates of *Staphylococcus aureus*. Antimicrob. Agents Chemother. 49, 2098–2101 10.1128/AAC.49.5.2098-2101.2005 15855537 PMC1087674

[B78] Tsaneva I. R. Muller B. West S. C. (1992). ATP-dependent branch migration of Holliday junctions promoted by the RuvA and RuvB proteins of *E. coli*. Cell 69, 1171–1180 10.1016/0092-8674(92)90638-S 1617728

[B79] Ubeda C. Tormo M. A. Cucarella C. Trotonda P. Foster T. J. Lasa I. (2003). Sip, an integrase protein with excision, circularization and integration activities, defines a new family of mobile *Staphylococcus aureus* pathogenicity islands. Mol. Microbiol. 49, 193–210 10.1046/j.1365-2958.2003.03577.x 12823821

[B80] Uranga L. A. Balise V. D. Benally C. V. Grey A. Lusetti S. L. (2011). The *Escherichia coli* DinD protein modulates RecA activity by inhibiting postsynaptic RecA filaments. J. Biol. Chem. 286, 29480–29491 10.1074/jbc.M111.245373 21697094 PMC3190988

[B83] Viana D. Blanco J. Tormo-Mas M. A. Selva L. Guinane C. M. Baselga R. (2010). Adaptation of *Staphylococcus aureus* to ruminant and equine hosts involves SaPI-carried variants of von Willebrand factor-binding protein. Mol. Microbiol. 77, 1583–1594 10.1111/j.1365-2958.2010.07312.x 20860091

[B81] Wyatt M. D. Allan J. M. Lau A. Y. Ellenberger T. E. Samson L. D. (1999). 3-methyladenine DNA glycosylases: structure, function, and biological importance. Bioessays 21, 668–676 10440863 10.1002/(SICI)1521-1878(199908)21:8<668::AID-BIES6>3.0.CO;2-D

[B82] Zabicka D. Mlynarczyk A. Windyga B. Mlynarczyk G. (1993). Phage-related conversion of enterotoxin A, staphylokinase and beta-toxin in *Staphylococcus aureus*. Acta Microbiol. Pol. 42, 235–241 7516614

