# Supplementary material for: Chromosomal islands of Streptococcus pyogenes and related streptococci: molecular switches for survival and virulence
Source: Front Cell Infect Microbiol. 2014 Aug 12;4:109. doi: 10.3389/fcimb.2014.00109 (PMC4129442; doi:10.3389/fcimb.2014.00109)
Supplement: Supplemental Figure S1 — Identity Matrix of Integrases. An amino acid sequence identity matrix of the CI integrase proteins from streptococcus species that target mutL is presented. SpyCI integrases show a high degree of similarity with each other, as well as strong similarities to ones found in S. canis and S. dysgalactiae subspecies equisimilis. High amino acid similarities suggest that core sequences are conserved and required for integration into mutL. [file Presentation1.PDF]

	SanCI CCUG 39159	SanCI F0211	SanCI J4206	ScaCI FSL Z3-227	SeqCI SK1249	SinCI F0413	SinCI JTH08	SinCI SK54	SpaCI KCTC 11537	SpyCIM1	SpyCIM2	SpyCIM28	SpyCIM4	SpyCIM49	SpyCIM5	SpyCIM53	SpyCIM59	SpyCIM59.1	SpyCIM6	SpyCIM98.1
SanCI CCUG 39159		0.01	0.01	0.36	0.36	0.01	0.31	0.31	0.35	0.37	0.36	0.36	0.36	0.37	0.35	0.37	0.37	0.37	0.36	0.37
SanCI F0211	0.01		0	0.37	0.37	0.01	0.31	0.31	0.35	0.37	0.37	0.37	0.37	0.37	0.36	0.37	0.37	0.38	0.37	0.37
SanCI J4206	0.01	0		0.37	0.37	0.01	0.31	0.31	0.35	0.37	0.37	0.37	0.37	0.37	0.36	0.37	0.37	0.38	0.37	0.37
ScaCI FSL Z3-227	0.36	0.37	0.37		0.04	0.37	0.33	0.33	0.39	0.04	0.04	0.04	0.04	0.04	0.05	0.04	0.04	0.05	0.04	0.04
SeqCI SK1249	0.36	0.37	0.37	0.04		0.37	0.36	0.36	0.41	0.00	0	0	0.01	0.00	0.01	0.00	0.01	0.01	0.00	0.01
SinCI F0413	0.01	0.01	0.01	0.37	0.37		0.31	0.31	0.35	0.37	0.37	0.37	0.37	0.37	0.36	0.37	0.37	0.38	0.37	0.37
SinCI JTH08	0.31	0.31	0.31	0.33	0.36	0.31		0	0.38	0.36	0.36	0.36	0.35	0.36	0.35	0.36	0.36	0.36	0.35	0.36
SinCI SK54	0.31	0.31	0.31	0.33	0.36	0.31	0		0.38	0.36	0.36	0.36	0.35	0.36	0.35	0.36	0.36	0.36	0.35	0.36
SpaCI KCTC 11537	0.35	0.35	0.35	0.39	0.41	0.35	0.38	0.38		0.41	0.41	0.41	0.41	0.41	0.41	0.41	0.41	0.41	0.41	0.41
SpyCIM1	0.37	0.37	0.37	0.04	0.00	0.37	0.36	0.36	0.41		0.00	0.00	0.01	0	0.01	0	0.01	0.02	0.01	0.01
SpyCIM2	0.36	0.37	0.37	0.04	0	0.37	0.36	0.36	0.41	0.00		0	0.01	0.00	0.01	0.00	0.01	0.01	0.00	0.01
SpyCIM28	0.36	0.37	0.37	0.04	0	0.37	0.36	0.36	0.41	0.00	0		0.01	0.00	0.01	0.00	0.01	0.01	0.00	0.01
SpyCIM4	0.36	0.37	0.37	0.04	0.01	0.37	0.35	0.35	0.41	0.01	0.01	0.01		0.01	0.01	0.01	0.01	0.01	0.00	0.00
SpyCIM49	0.37	0.37	0.37	0.04	0.00	0.37	0.36	0.36	0.41	0	0.00	0.00	0.01		0.01	0	0.01	0.02	0.01	0.01
SpyCIM5	0.35	0.36	0.36	0.05	0.01	0.36	0.35	0.35	0.41	0.01	0.01	0.01	0.01	0.01		0.01	0.01	0.02	0.01	0.01
SpyCIM53	0.37	0.37	0.37	0.04	0.00	0.37	0.36	0.36	0.41	0	0.00	0.00	0.01	0	0.01		0.01	0.02	0.01	0.01
SpyCIM59	0.37	0.37	0.37	0.04	0.01	0.37	0.36	0.36	0.41	0.01	0.01	0.01	0.01	0.01	0.01	0.01		0.01	0.00	0.01
SpyCIM59.1	0.37	0.38	0.38	0.05	0.01	0.38	0.36	0.36	0.41	0.02	0.01	0.01	0.01	0.02	0.02	0.02	0.01		0.01	0.01
SpyCIM6	0.36	0.37	0.37	0.04	0.00	0.37	0.35	0.35	0.41	0.01	0.00	0.00	0.00	0.01	0.01	0.01	0.00	0.01		0.00
SpyCIM98.1	0.37	0.37	0.37	0.04	0.01	0.37	0.36	0.36	0.41	0.01	0.01	0.01	0.00	0.01	0.01	0.01	0.01	0.01	0.00	
