# Supplementary material for: Chromosomal islands of Streptococcus pyogenes and related streptococci: molecular switches for survival and virulence
Source: Front Cell Infect Microbiol. 2014 Aug 12;4:109. doi: 10.3389/fcimb.2014.00109 (PMC4129442; doi:10.3389/fcimb.2014.00109)
Supplement: Supplementary file 2 [file DataSheet1.DOC]

**Supplemental Table 1. Streptococcal species that are grouped by either the presence or absence of MDR gene *lmrP.***

|  |  | **Streptococcal species** | **CI*** |
| --- | --- | --- | --- |
| **Species with *lmrP*** | | | |
|  | Group A | *S. pyogenes* | SpyCI (*mutL*) |
|  | Group B | *S. agalactiae* | SagCI (*rpsD*) |
|  | Group C | *S. equi* sub. *zooepidemicus* |  |
|  |  | *S. dysgalactiae* sub. *equisimilis* | SeqCI (*mutL*) |
|  |  | *S. dysgalactiae* sub. *dysgalactiae* |  |
|  | Group D | *S. entericus* |  |
|  | Group G | *S. canis* | ScaCI (*mutL*) |
|  | Viridans group | *S. oralis* |  |
|  |  | *S. tigurinus* |  |
|  |  | *S. massiliensis* |  |
|  | Other species | *S. iniae* |  |
|  |  | *S. uberis* |  |
|  |  | *S. parauberis* | SpaCI (*mutL*) |
|  |  | *S. didelphis* |  |
|  |  | *S. porcinus* |  |
|  |  | *S. pseudoporcinus* |  |
|  |  | *S. ovis* |  |
|  |  | *S. thoraltensis* |  |
| **Species without *lmrP*** | | | |
|  | Group D | *S. bovis* |  |
|  |  | *S. gallolyticus* subsp. *gallolyticus* |  |
|  | Milleri group | *S. anginosus* | SanCI (*mutL*) |
|  |  | *S. intermedius* | SinCI (*mutL*) |
|  |  | *S. constellatus* |  |
|  | Viridans group | *S. mitis* |  |
|  |  | *S. pneumoniae* | SpnCI (*uvrA*) |
|  |  | *S. mutans* |  |
|  | Salivarius group | *S. thermophilus* | SthCI (*metE*) |
|  | Sanguinis group | *S. sanguinis* |  |
|  | Suis group | *S. suis* | SsuCI (*recF*) |

*CI: chromosomal island and attachment site (*attB*)
