# Supplementary material for: Chromosomal islands of Streptococcus pyogenes and related streptococci: molecular switches for survival and virulence
Source: Front Cell Infect Microbiol. 2014 Aug 12;4:109. doi: 10.3389/fcimb.2014.00109 (PMC4129442; doi:10.3389/fcimb.2014.00109)
Supplement: Supplementary file 3 [file DataSheet2.DOC]

**Supplemental Table 2. Identification of the phylogenetic tree branches presented in Fig. 3.**  The attachment site for all is *mutL* except where noted in parenthesis.

| **No.** | **Identification** | **No.** | **Identification** |
| --- | --- | --- | --- |
| 1 | SpyCIM5 Manfredo | 25 | SpaCI KCTC 11537 |
| 2 | SpyCIM49 GA40634 | 26 | CI from uncharacterized streptococcus species M334 (*uvrA*) |
| 3 | SpyCIM4 | 27 | SmiCI 11/5 (*uvrA*) |
| 4 | SpyCIM5 UTMEM-1 | 28 | SpsCI ATCC BAA-960 (*uvrA*) |
| 5 | SpyCIM28 | 29 | SmiCI SK1073 (*uvrA*) |
| 6 | SpyCIM2 | 30 | SmiCI B6 (*manA*) |
| 7 | SpyCIM5 UTSW-2 | 31 | SpnCI GA17719 |
| 8 | SpyCIM5 GA40377 | 32 | SpnCI Hungary 19A-6 |
| 9 | SeqCI 167 | 33 | SpnCI 2080913 |
| 10 | SpyCIM5 GA41046 | 34 | SpnCI GA14688 |
| 11 | SpyCIM1 | 35 | SpnCI 2071004 |
| 12 | SpyCIM53 | 36 | SmiCI SK616 (*uvrA*) |
| 13 | SeqCI SJ1249 | 37 | SmiCI SK569 |
| 14 | SpyCIM49 GA16797 | 38 | SmiCI SK1080 (*urvA*) |
| 15 | SpyCIM59.1 | 39 | SinCI ATCC 27335 |
| 16 | SpyCIM49 GA03747 | 40 | SinCI JTH08 |
| 17 | ScaCI FSLZ3-227 | 41 | SinCI SK54 |
| 18 | SpyCIM59 | 42 | SinCI F0413 |
| 19 | SpyCIM6 GA19700 | 43 | SanCI whileyi MAS624 |
| 20 | SpyCIM6 | 44 | SanCI whileyi CCUG39159 |
| 21 | SpyCIM6 GA41039 | 45 | SanCI C238 |
| 22 | SpyCIM6 GA41208 | 46 | SanCI 1505 |
| 23 | SpaCI KRS-02083 | 47 | SanCI J4206 |
| 24 | SpaCI KRS-02109 | 48 | SanCI F0211 |
